# Professional Notes

**Published:** 1888-01-07

**Authors:** Geo W. Potter


### PROFESSIONAL NOTES.

BY GEO. W. POTTER, M.D.

APPENDICITIS or inflammation of the vermiform appendix is a disease with which the ordinary practitioner has few opportunities of becoming familiar. It is one in which errors of diagnosis are easy, and perhaps frequent; and such errors may result in the loss of many lives. A skilful and conscientious medical man is always prompt to take alarm at new and unusual symptoms. The diseases ordinarily met with in general practice are such as are easily recognised; but now and again unlooked for symptoms manifest themselves, and it is these which test the natural capacity and judgment of the physician, as well as his technical skill. Many unfamiliar diseases are such as are peculiar to certain climates, or are only to be met with under particular circumstances, and the practitioner, whose work lies in different climates, or in districts remote from those particular circumstances may be excused if he pay no special attention to them. But every man has a vermiform appendix, which may develop inflammation at home as well as abroad, in the country or in the town, in the poor man's abdominal cavity, or in his richer neighbour's. Any practitioner may be called upon at a moment's notice to deal with a case of appendicitis in the workhouse or in the squire's hall. A brief discussion of the disease, its symptoms and its treatment, will not be considered out of place.

---

DR. REGINALD H. FITZ, Shattuck Professor of Pathological Anatomy in Harvard University, has treated the subject of appendicitis at some length, comparing it especially with typhlitis and perityphlitis. In the brief space at disposal here all that can be attempted is to point out the principal differentiating characteristics of the disease, and to indicate what seem to be the soundest principles of treatment. Dr. Fitz is of opinion that the diagnosis is not very difficult, and probably this may be so in the case of physicians of large experience in various kinds of practice. Those of smaller personal knowledge would perhaps prefer not to have their skill put to too severe a test. An important point to be noticed is that the symptoms may be *latent* for a considerable period after the inflammatory process has commenced. "This latency . . . is such that the eventual diagnosis is obscured, and the desirable method of treatment hopelessly postponed. . . . The presence, therefore, of the symptoms now to be mentioned, in individuals from whom the history of one, and particularly of several such attacks is to be obtained, is of marked importance in aiding diagnosis. . . *Sudden, severe abdominal pain* is the most constant first decided symptom of perforating inflammation of the appendix. . . The pain is usually intense . . . and may be accompanied by a chill, or nausea and vomiting. . . *Fever is the next constant symptom.* The temperature is seldom very high, ranging usually from 100 deg. F. to 102 deg. F. The maximum recorded in Fitz's cases is 103·5 deg. F. . . *A circumscribed swelling in the right iliac fossa is the third characteristic feature.* "This symptom, when present, is evidently of the utmost value in diagnosis, as its appropriate treatment most favourably modifies the prognosis. Errors in the diagnosis of appendicitis have been numerous, chiefly because the *cardinal symptoms of localised pain, general heat, and circumscribed swelling have not been duly appreciated in their defined sequence.*" Of course a differential diagnosis is to be made; and the experienced practitioner will readily recall to mind those affections with which this lesion may be confounded, as, for example, typhlitis, perityphlitis, colic, the passing of calculi, general peritonitis, strangulated hernia, movable kidney, and the like. If, however, the three "*cardinal points*" of Fitz be carefully borne in mind there should be no insuperable difficulty in arriving at a correct conclusion.

---

THE TREATMENT OF APPENDICITIS demands the most thoughtful and conciencious consideration. We have first of all to make for ourselves a mental picture of the actual condition of the appendix and surrounding parts. Not only so, but we must be able to understand also what to expect as the disease progresses; and when to expect it. The inflammation may at first be "simple," though due probably to some offending cause—as, for example, a seed, or a portion of the inspissated contents of the bowel, or a blow. If the offending cause could be removed the simple inflammation would tend naturally to resolution and recovery of the patient. But how is it to be removed? As a matter of fact it frequently is not removed; and the inflammation progresses to suppuration, the suppuration to perforation, the perforation to general peritonitis, and the peritonitis to death; and death takes place generally within a few days of the first manifestation of the symptoms. That is a chain of sequence which should always be present to the mind of the practitioner from the moment the diagnosis is made. Here we are then in the presence of a patient who, according to our best judgment, is suffering from definite inflammation of the vermiform appendix. What are we going to do for the helpless man in his deadly peril? We *must* lay down a line for *general* treatment; we *may* also decide upon some *local* attempt. For the general treatment, "to keep the bowels quiet should be the first and last thought," says Dr. Fitz. "Absolute rest in bed," he continues, "liquid diet in small quantities often repeated, and, *above all, sufficient opium to neutralize pain.* A sufficiency may seem enormous. Pétrequin gave a grain of opium every hour till the pain was relieved, with the result of administering 107 grains in six days. Clarke gave a boy, fourteen years old, 1,350 drops of laudanum in one day. A cathartic or laxative may be demanded by the patient or friends, and an enema may be thought desirable as a diagnostic aid. *It is to be remembered that these may be the means of at once exciting a general peritonitis.* In the milder cases the pain disappears in a few days, vomiting ceases, and within five or six days tenderness and distention disappear. The bowels open spontaneously a few days after the discontinuance of the opium. They may remain bound for twenty-four days, yet the general health need not suffer." About a fourth of the cases may be expected to terminate in resolution and for these the general treatment indicated is all that is required. What is to be done for the other three-fourths? "Operative interference," says Fitz, "is demanded in two-thirds of all the cases." A question of primary importance must here be asked, viz., What is the object aimed at by the operation? It is the prevention of perforation and general peritonitis, circumstances which are practically always fatal. Fitz lays down this rule: "If, after the first twenty-four hours from the onset of the severe pain, the peritonitis is evidently spreading and the condition of the patient is grave, the question should be entertained of an *immediate operation for exposing the appendix and determining its condition with reference to its removal.*" That is the early operation. But "if surgical interference is not instituted within the first twenty-four hours after the onset of the sudden and intense right iliac pain, to keep the bowels quiet must still be the injunction. The formation of the tumour, the circumscribing of the peritonitis is then to be awaited. It is sure to form in the large majority of cases if the patient lives long enough. In more than two-thirds of the cases the contents will escape externally or internally. *Without surgical aid the escape is into the peritonal cavity in most instances, with a rapidly fatal result.* . . . Willard Parker demonstrated the success of this operation (the opening of the abdomen) in three out of four cases, and it is his advocacy of an early operation which has produced such favourable results since 1867. . . . He considered that *an incision made between the fifth and twelfth days was practicable, safe, and justifiable.* Even when the diagnosis was doubtful if no abscess had formed, in case one should be in process of formation, an external opening would tend to make it point in a safe direction, and if no abscess should form, a free incision would relieve tension." Early as this date (the fifth day) may seem for an operation, Dr. Fitz is of opinion that "if the indications for operating justified the election of a date as early as the fifth day, they still more justify the choice of the third day." This conclusion seems eminently reasonable in view of the extreme and undeniable risks of delay.

